# Repetitive syncope in a newborn leading to pacemaker implantation: Evidence for dopamine beta-hydroxylase deficiency

**DOI:** 10.1016/j.hrcr.2025.03.026

**Published:** 2025-05-09

**Authors:** Nathan Marimpouy, Grégoire de la Villéon, Leslie Placide, Laura Delsarte, Mathieu Granier, François Massin, Bertrand Léobon, Abdullah Almadhaani, Albano C. Meli, Marie Vincenti, Jean-Luc Pasquié

**Affiliations:** 1 Department of Cardiology, Electrophysiology and Cardiac Stimulation, Hôpital Arnaud de Villeneuve, Montpellier University Hospital, Montpellier, France; 2 Department of Pediatrics, Hôpital Arnaud de Villeneuve, Montpellier University Hospital, Montpellier, France; 3 PhyMedExp, University of Montpellier, Inserm, CNRS, CHU Montpellier, Montpellier, France; 4 Department of Pediatric Cardiac Surgery, Hôpital Haut Leveque, CHU Bordeaux, Pessac, France

**Keywords:** Atrioventricular block, Autonomous nervous system, Cardiac pacing, Catecholamine, Dopamine beta-hydroxylase deficiency, Genetic disorder, L-threo-dihydroxyphenylserine, Newborn, Syncope

**Key Teaching Points**

• Dopamine beta-hydroxylase (DBH) deficiency is a very rare condition, characterized mainly by severe and early orthostatic hypotension. Hypothermia and hypoglycemia may occur during the neonatal period. Rarely, bradycardia may occur, leading to syncope.
• DBH deficiency is caused by mutations in the *DBH* gene (9q34), which codes for the enzyme DBH, which converts dopamine into noradrenaline.
• This results in undetectable levels of noradrenaline and adrenaline and very high levels of dopamine in the plasma, urine, and cerebrospinal fluid.
• Precursor treatment with L-threo-dihydroxyphenylserine (droxidopa) gives excellent clinical and long-term results.

## Introduction

Dopamine beta-hydroxylase (DBH) deficiency is a rare congenital autosomal recessive disorder, with an estimated prevalence of fewer than 1 in 1,000,000 individuals in the general population. DBH is an enzyme responsible for converting dopamine into norepinephrine, which is subsequently transformed into epinephrine.^1^ A deficiency in this enzyme leads to nearly undetectable levels of norepinephrine and epinephrine in the plasma, accompanied by significantly elevated dopamine levels. The primary symptoms are related to dysautonomia and include severe orthostatic hypotension, eyelid ptosis, and profound hypoglycemia. The clinical presentation of DBH deficiency is often atypical, leading to frequent delays in diagnosis, which is typically made during adolescence or early adulthood. Although several cases have been reported in recent years, most were diagnosed at an advanced age.^2^ We recently identified a case in a newborn with a history of recurrent malignant syncope, necessitating cardiac pacing. The diagnosis of DBH deficiency was made later, and the symptoms were successfully managed with L-threo-dihydroxyphenylserine (L-DOPS).

## Case report

**Figure 1:**
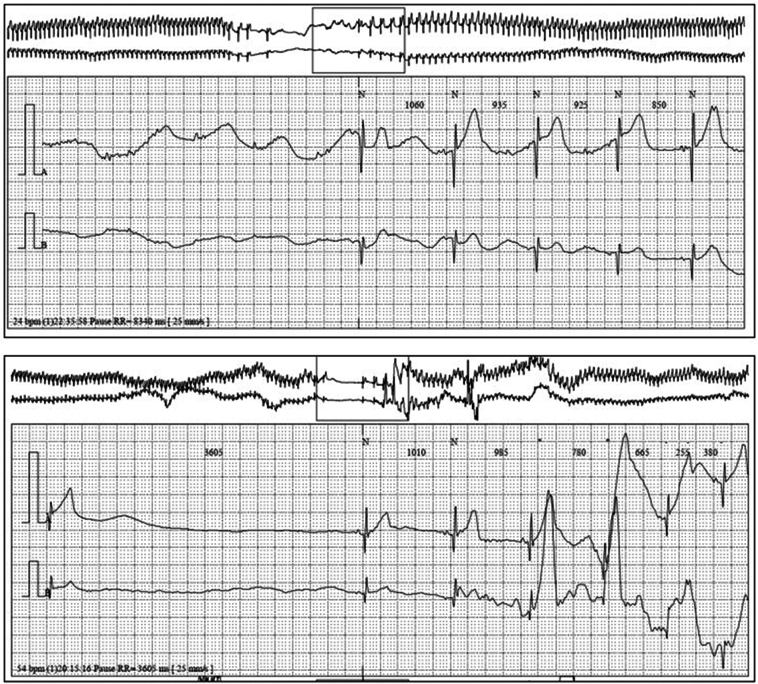
24-hour Holter electrocardiogram demonstrating episodes of sinus node dysfunction and atrioventricular block.

A premature female newborn was delivered via cesarean section at 26 weeks of gestation owing to profound bradycardia and intrauterine growth restriction during pregnancy. In the early postnatal period, she exhibited episodes of faintness and sudden loss of consciousness associated with severe bradycardia. Rhythm monitoring revealed high-grade atrioventricular block and sinus node dysfunction (SND), leading to significant asystoles, often lasting up to 10 seconds. These episodes were preceded by progressively increasing RR intervals and were documented on repeated 24-hour Holter electrocardiogram (Figure 1). Baseline electrocardiogram showed sinus rhythm without conduction abnormalities, with a heart rate of 110 beats per minute, which is slightly low for her age. Transthoracic echocardiography ruled out any underlying structural heart disease. Hypertonic vagal tone and sleep apnea (confirmed by polysomnography) were suspected as contributing factors, and the newborn was discharged at 39 weeks of gestation.

**Figure 2:**
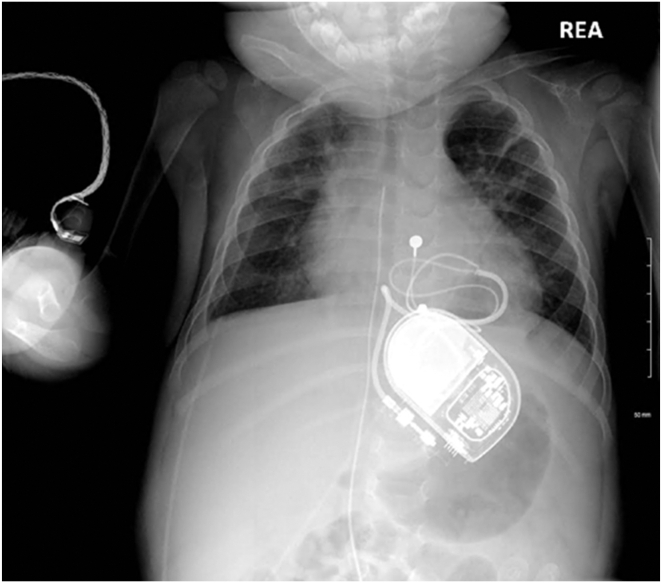
Chest radiograph showing the single-chamber ventricular epicardial lead and pacemaker device.

During follow-up, recurrent syncopal episodes were observed, leading to hospitalizations at 1 and 2 months of age owing to SND coinciding with viral bronchiolitis (caused by rhinovirus and parainfluenza). These episodes were attributed to a hypertonic vagal response secondary to viral infection. After the second episode, treatment with disopyramide was initiated to alleviate symptoms, leveraging its anticholinergic properties. At 8 months of age, she experienced severe syncope accompanied by profound bradycardia during another respiratory viral infection. After a multidisciplinary team discussion, a decision was made to implant an epicardial single-chamber cardiac pacemaker (Biotronik Edora 8 SR-T with a Medtronic 4968 bipolar steroid-eluting lead) (Figure 2). After implantation, no further syncopal episodes occurred.

**Figure 3:**
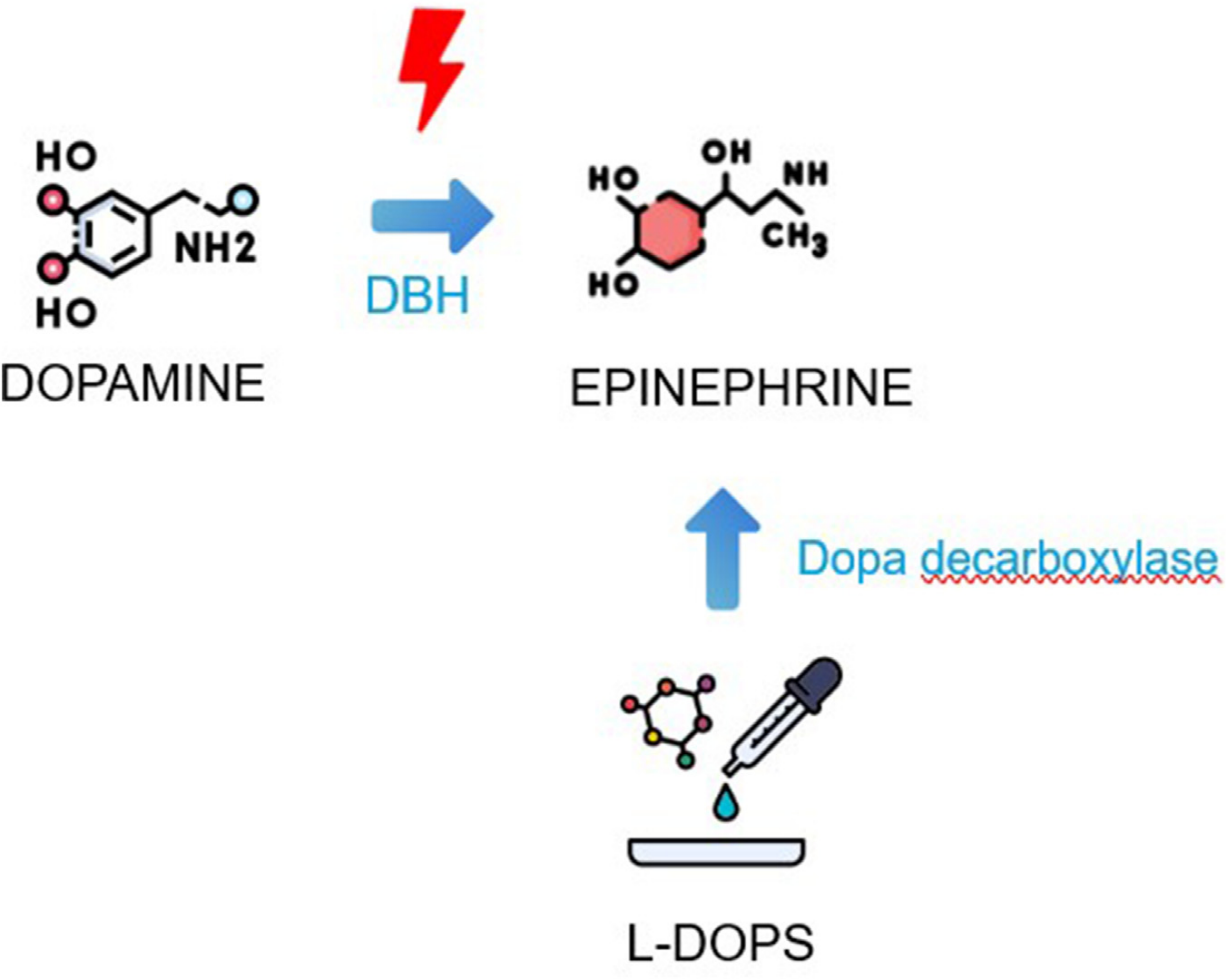
Alternative pathway of droxidopa supplementation.^3^ DBH = Dopamine beta-hydroxylase; L-DOPS = L-threo-dihydroxyphenylserine.

At the age of 3 years, she was admitted for dizziness, and blood tests revealed profound hypoglycemia (0.25 g/L on capillary glucose measurement). Home monitoring of capillary glucose levels showed recurrent hypoglycemia, and further investigations identified transient hyperlactacidemia. Plasma catecholamine levels were notably low (metanephrine < 10 ng/L, normal < 181 ng/L; normetanephrine < 20 ng/L, normal < 236 ng/L), whereas the dopamine-to-creatinine ratio was at the upper limit of normal (599 μmol/mmol, normal < 740 μmol/mmol). Genetic screening identified a pathogenic homozygous variant in the *DBH* gene (c.339+2T>C), confirming DBH deficiency. Supplementation with L-DOPS effectively alleviated her symptoms, and no further syncopal episodes were reported (Figure 3). Long-term remote monitoring of the device revealed a 0% pacing rate, indicating no reliance on the pacemaker for cardiac rhythm support.^3^

## Discussion

To the best of our knowledge, this is the youngest published case of DBH deficiency. The presentation, characterized by prolonged periods of asystole associated with syncope, is also highly unusual.

**Figure 4:**
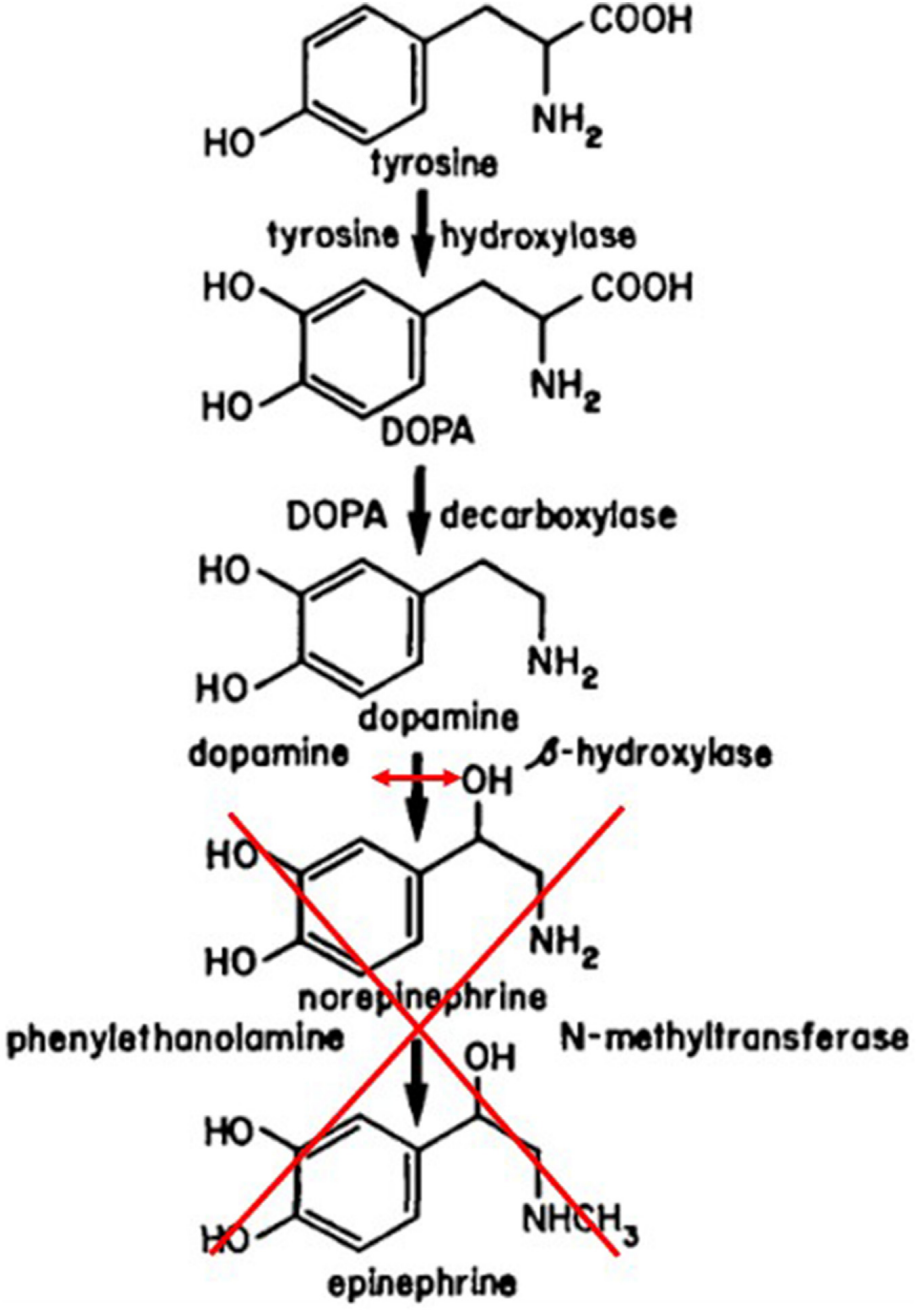
Role of dopamine beta-hydroxylase in catecholamine synthesis. DOPA = dihydroxyphenylalanine.

DBH deficiency is a rare congenital disorder often diagnosed late owing to its polymorphic clinical presentation (Figure 4). DBH is an enzyme that converts dopamine into norepinephrine, which is further metabolized into epinephrine.^1^ Its deficiency results in nearly undetectable plasma levels of norepinephrine and epinephrine, alongside elevated dopamine levels. Although various symptoms have been described in the literature,^1^^,^^2^ orthostatic hypotension, hypoglycemia, and eyelid ptosis seem to be the most prevalent. In this case, recurrent hypoglycemia was the key clue that led to the suspicion of DBH deficiency. The only known effective treatment is supplementation with L-DOPS (droxidopa), which uses an alternative pathway for catecholamine synthesis.^3^ After initiation of L-DOPS, the patient experienced no further symptoms, and remote monitoring showed a 0% ventricular pacing rate, indicating resolution of bradycardia.

In this case, cardiac pacing was implemented after a multidisciplinary heart team discussion owing to multiple symptomatic and debilitating syncopal episodes prior to diagnosis. However, the remote monitoring data suggest that pacing might have been avoidable. That said, the possibility of an underlying conduction disorder and its progression cannot be entirely ruled out.

Interestingly, in our case, syncope episodes predominantly occurred at rest and in the supine position, suggesting that hypotension was not the primary mechanism, unlike in typical cases of DBH deficiency where orthostatic hypotension is a common cause of syncope. The pathophysiology may instead involve the Bezold-Jarisch reflex, which originates from inhibitory cardiac sensory receptors and is mediated by nonmyelinated C fibers, as described by Mark.^4^ Under normal conditions, these receptors reduce their activity during orthostatic stress or hypovolemia, leading to a compensatory increase in heart rate. However, in cases of severe hypovolemia and hypotension, vigorous contraction of an underfilled heart may paradoxically stimulate these nonmyelinated fibers, resulting in reflex bradycardia and vasodilation. This mechanism could explain the episodes of SND and atrioventricular block observed in our patient.

Sun and colleagues^5^ investigated the role of the *DBH* gene in cardiac conduction tissue development during embryogenesis. In *Dbh* knockout mice, abnormalities in conduction tissue were observed, including prolonged refractory periods and lower Wenckebach and 2:1 conduction rate at slower heart rates. We hypothesize that the homozygous catecholamine deficiency in our case may explain both the unusually young age of presentation and the severity of the disease, which affected the sinus and atrioventricular nodes. A primary dysfunction in the development or migration of conduction tissue owing to *DBH* gene deficiency could also be a contributing factor.

The use of disopyramide, with its anticholinergic properties, may have contributed to symptom resolution by counterbalancing the catecholamine deficiency. This effect has been studied in patients prone to transient hypotension and bradycardia reflex, where it was shown to attenuate the exaggerated neural reflex originating from cardiac sensory receptors.^6^

## Conclusion

This case represents the youngest diagnosis of DBH deficiency reported to date. DBH deficiency remains a rare and often underrecognized condition, typically diagnosed late in life. The cornerstone of treatment is supplementation with L-DOPS, which restores catecholamine synthesis. Clinicians should consider DBH deficiency as a potential, albeit unusual, cause of syncope in children.

Emerging fundamental research suggests that the *DBH* gene plays a role in the development of cardiac conduction cells, with studies in mice indicating potential conduction abnormalities associated with its deficiency. Long-term follow-up of patients with DBH deficiency could provide valuable insights into the progression of conduction disorders over their lifetime, potentially linked to developmental abnormalities of the conduction tissue during embryogenesis.

## Disclosures

The authors have no conflicts of interest to disclose.
